# Protein Network Analysis of Whole Exome Sequencing of Severe Preeclampsia

**DOI:** 10.3389/fgene.2021.765985

**Published:** 2022-06-02

**Authors:** Jessica Schuster, George A. Tollefson, Valeria Zarate, Anthony Agudelo, Joan Stabila, Ashok Ragavendran, James Padbury, Alper Uzun

**Affiliations:** ^1^ Pediatrics, Women and Infants Hospital, Providence, RI, United States; ^2^ Pediatrics, Warren Alpert Medical School, Brown University, Providence, RI, United States; ^3^ Center for Computation and Visualization, Brown University, Providence, RI, United States; ^4^ Computational Biology of Human Disease, Brown University, Providence, RI, United States; ^5^ Center for Computational Molecular Biology, Brown University, Providence, RI, United States

**Keywords:** preeclampsia, complex diseases, network biology, protein protein interaction (PPI), computational biology

## Abstract

Preeclampsia is a hypertensive disorder of pregnancy, which complicates up to 15% of US deliveries. It is an idiopathic disorder associated with several different phenotypes. We sought to determine if the genetic architecture of preeclampsia can be described by clusters of patients with variants in genes in shared protein interaction networks. We performed a case-control study using whole exome sequencing on early onset preeclamptic mothers with severe clinical features and control mothers with uncomplicated pregnancies between 2016 and 2020. A total of 143 patients were enrolled, 61 women with early onset preeclampsia with severe features based on ACOG criteria, and 82 control women at term, matched for race and ethnicity. A network analysis and visualization tool, Proteinarium, was used to confirm there are clusters of patients with shared gene networks associated with severe preeclampsia. The majority of the sequenced patients appear in two significant clusters. We identified one case dominant and one control dominant cluster. Thirteen genes were unique to the case dominated cluster. Among these genes, LAMB2, PTK2, RAC1, QSOX1, FN1, and VCAM1 have known associations with the pathogenic mechanisms of preeclampsia. Using bioinformatic analysis, we were able to identify subsets of patients with shared protein interaction networks, thus confirming our hypothesis about the genetic architecture of preeclampsia.

## Introduction

Preeclampsia is a hypertensive disorder of pregnancy. It is associated with a higher risk of hypertension and cardiovascular disease later in life. Women who had preeclampsia have a twofold increased risk of death from cardiovascular diseases (Bokslag et al., 2016; Neerukonda et al., 2020). There is evidence that preeclampsia originates in part from genetic causes that include contributions from the maternal, paternal and fetal genome (Zusterzeel et al., 2002; Cnattingius et al., 2004; Nilsson et al., 2004; Kobayashi, 2015; Than et al., 2018). The heritability of preeclampsia is up to 52% (Salonen Ros et al., 2000; Chappell and Morgan, 2006). The role of genetics in preeclampsia is also supported by family-based observations (Chappell and Morgan, 2006; Nejatizadeh et al., 2008) with more than 100 studies showing a 2- to 5-fold increased risk among family members of affected women (Chesley et al., 1968; Sutherland et al., 1981; Arngrimsson et al., 1990; Cincotta and Brennecke, 1998; Mutze et al., 2008; Ward, 2008). The recurrence risk for preeclampsia in the daughters of either eclamptic or preeclamptic patients is 20–40% (Serrano, 2006; Genc and Schantz-Dunn, 2007). Nonetheless, there is no current consensus among the published results in regards to associated genes and the pathogenesis of the disease.

The genetic basis for complex diseases involves the interaction of multiple genes in discrete networks and pathways (Loscalzo et al., 2007). Although complex diseases show increased recurrence risk in families, they do not follow a simple Mendelian pattern of inheritance (Smith and Ebrahim, 2003). Computational methods have been used to analyze networks of genes and to find biological subnetworks due to the genetic heterogeneity of e diseases that are linked to a variety of disorders (Wall et al., 2009). There are several studies employing computational methods to identify important genes associated with hypertension. Ran et al. analyzed the protein protein interaction (PPI) network topology and molecular connectivity between protein pathways to identify associations with hypertension (Ran et al., 2013). Researchers have also developed a machine-learning algorithm to predict novel hypertension associated genes (Li et al., 2017).

We hypothesize that the genetic architecture of complex diseases like preeclampsia is described by clusters of patients with variants in genes in shared PPI networks. We sought to test this hypothesis using whole exome sequencing in carefully selected patients with early onset preeclampsia with severe clinical features**.** We compared variants identified in women with early onset, idiopathic severe preeclampsia with term controls without personal or family history of pregnancy related hypertensive disorders. We built and implemented *Proteinarium,* a novel multi-sample PPI tool, to identify clusters of patients with shared PPI networks.

## Materials and Methods

### Study Population

Women & Infants Hospital of Rhode Island (WIH) is the only provider of high-risk perinatal services in Rhode Island, northeastern Connecticut and southeastern Massachusetts. We used this population-based service to enroll preeclamptic mothers with early onset, severe features, based on ACOG criteria, as well as term mothers with no history of preeclampsia (Roberts et al., 2013).

This case/control study was approved by the Institutional Review Board of WIH (Project ID: WIH 16–0031). Between the years 2016–2020, we reviewed the records of all early-onset preeclamptic mothers with severe features delivering <34 weeks. Following informed consent, we asked explicit questions about preeclampsia in mother, grandmother, first order relatives and also paternal relatives. Gestational age was determined by best obstetrical estimate. In almost all cases, this was by first trimester ultrasound. Clinical history, with an emphasis on additional risk factors including medical illnesses and drug use was recorded. Hypertensive disorders include a broad range of different phenotypes. Again, in order to leverage the likelihood of genetic discovery associated with preeclampsia, we excluded preeclamptic mothers with personal or family history of other hypertensive disorders. Controls were mothers who delivered ≥37 weeks’ gestation for whom the formal genetic interview revealed no history of preterm birth or pregnancy related hypertensive disorders on either the maternal or paternal side of the pedigree. A total of 143 patients were enrolled, 61 women with early onset preeclampsia with severe features, and 82 control women at term, matched for race and ethnicity.

### Whole Exome Sequencing

EDTA stabilized, residual maternal whole blood was obtained from each mother and stored at -80 °C. Samples were sent to an outside facility for whole exome sequencing that was blind to disease status. The library was sequenced on an Illumina HiSeq 4000 using 150 bp paired-end protocols.

### Sequence Data

For variant discovery we used the Gene Analysis Tool Kit (GATK) V4 to analyze the sequence reads (Van der Auwera et al., 2013). Haplotype Caller was applied for variant detection (Poplin et al., 2018). Variants were flagged as low quality and filtered using established metrics: if three or more variants were detected within 10bp; if four or more alignments mapped to different locations equally well; if coverage was less than ten reads; if quality score <30; if low quality for a particular sequence depth (variant confidence/unfiltered depth <1.5); and if strand bias was observed (Phred-scaled *p*-values using Fisher’s Exact Test >200).

### Genotype Testing

To identify variants that were differentially abundant between cases and controls, we used a Markov Chain Monte Carlo (MCMC) Fisher Exact Test to compare the frequency of the homozygous reference, homozygous alternative, and the heterozygous genotypes between cases and controls. Eigenstrat detected no significant population stratification (Price et al., 2006). None of the eigenvectors were associated with race/ethnicity.

### Univariate Analysis: Variant Annotation

We applied a strict filter-based annotation using ANNOVAR (Wang et al., 2010). We identified deleterious variants with Polyphen 2 HDIV, SIFT and CADD (Ng and Henikoff, 2003; Adzhubei et al., 2010; Kircher et al., 2014; Genomes Project et al., 2015). We used the following thresholds: Polyphen 2 HDIV prediction if a change is damaging (≥0.957), a SIFT score (<0.05), a CADD score >15, and minor allele frequency (MAF) < 0.05 from the 1,000 Genome Project (Genomes Project et al., 2015).

### Multivariate Protein Protein Interaction Analysis

We hypothesized that the genetic architecture underlying complex disorders is best explained by subsets of patients with variants in shared networks and pathways sufficient to express the phenotype. To analyze our whole exome sequencing data, we developed *Proteinarium,* a novel multi-sample PPI analysis and visualization tool (Armanious et al., 2020). For each sample, the top 60 genes, corresponding to the most significant, differentially abundant variants (ranked by genotype testing *p* value) were used as the seed genes for input into *Proteinarium*. The pipeline for *Proteinarium* is described in great detail in Armanious et al., 2020. In brief, these seed genes were used to create a PPI network for *each* patient. After generating the network graphs for each patient, *Proteinarium* calculates the similarity between each pair of graphs using the Jaccard distance. This similarity matrix is then used as the input to cluster the set of graphs. Clustering is performed hierarchically using Unweighted Pair Group Method with Arithmetic Mean (UPGMA). *Proteinarium* outputs the results of the clustering algorithm as a dendrogram and shared interaction network graphs. For each cluster, the abundance of cases is compared to that of the controls using Fisher’s exact test. Each cluster can be visualized as a layered graph of its constituent networks. *Proteinarium* was implemented with the minimum path length parameter set to 2, to include only those pathways in which seed proteins are connected directly to each other *and/or* via a single intermediary protein. We refer to these intermediary connecting proteins as imputed proteins.

### Network Separation Testing

Separation testing is a computational approach for determining the genetic similarity between diseases by comparing their protein protein interaction networks from the interactome (Menche et al., 2015). It compares the shortest distances between network proteins *within* each disease or network to the shortest distances *between* the disease networks. A positive separation score indicates that there is a physical separation between networks within the interactome. We computed the separation score between the layered network graphs of clusters identified as associated to a specific phenotype via the Fisher exact test by *Proteinarium* (Armanious et al., 2020).

## Results

The clinical characteristics and the race/ethnicity distribution of the patients are shown in Table 1. As can be seen, the gestational age at delivery, systolic blood pressure, frequency of proteinuria, impaired liver function, thrombocytopenia, cerebral visual symptoms and fetal growth retardation were all significantly different between the groups, which was expected by our definition of severe preeclampsia.

**TABLE 1:** Clinical characteristics of patients. Mean +SD.

Categories	Case (n = 61)	Control (n = 82)
Gestational age of delivery and life style		
*Age (mean)*	29.1 ± 5.0	29.4 ± 5.3
*Grava (mean)*	2.1 ± 1.2	2.5 ± 1.6
*Job strenuous (%)*	26.2%	28.0%
*Obesity (%)*	31.1%	23.1%
Race/Ethnicity		
African_American (%)	9.8%	4.8%
Asian (%)	3.2%	3.6%
Caucasian (%)	55.7%	56.1%
Hispanic (%)	22.9%	28.0%
Native_American (%)	1.6%	1.2%
Other_Racial_ID (%)	6.5%	6.1%
Abnormal laboratory values		
Systolic_bp (mean, mmHg)	170.8 ± 14.4	117.6 ± 9.6
Proteinuria (%)	65.5%	0.00%
Impaired_liver_function (%)	55.7%	2.4%
Thrombocytopenia (%)	14.7%	0.0%
Cerebral_visual_symptoms (%)	55.7%	0.0%

**FIGURE 1:**
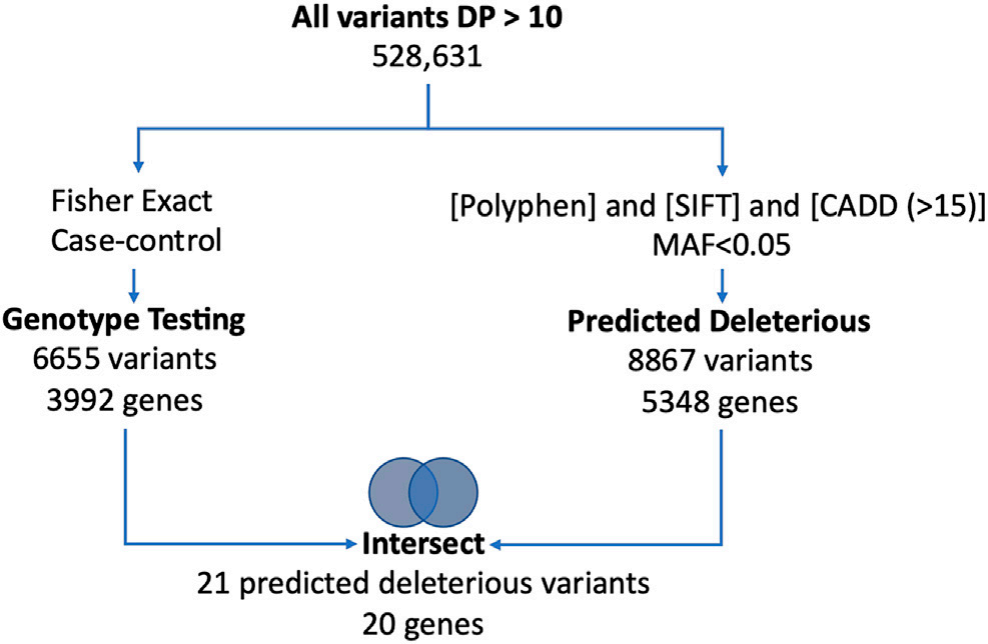
Figure shows the univariate work flow for analysis of the whole exome sequencing results.

High quality sequence data with a Phred score ≥30 from well-balanced pools with over 19, 000, 000 reads/patient, 40X average depth of coverage, with more than 80% of sequence reads with at least 20X coverage were observed. We identified a total of 528,630 variants including 187,915 exonic variants. The work flow for the univariate analysis is shown in Figure 1. After application of the initial filters for coverage and variant pathogenicity, there were 8,868 predicted deleterious variants (available at **Online_Supplementary Table S1** ). Among these, 21 variants were nominally associated with preeclampsia by genotype testing. All were non-synonymous, exonic variants (Table 2). Nonetheless, none of these variants met genome-wide significance after correction for multiple comparison testing.

**TABLE 2:** Pathogenic, nominally significant (based on genotype testing, *p* < 0.05) gene variants identified by univariate analysis. Genomic positions are based on Human Feb. 2009 (GRCh37/hg19) Assembly. *p* value represents the genotype testing.

Chr	Pos	Gene	HGNC ID	SNP	Polyphen2_HDIV	SIFT	CADD	MAF cases (in the cohort)	MAF controls (in the cohort)>	*p* value
1	97,770,920	DPYD	3012	rs1801160	0.998	0	23.5	0.010	0.049	0.032
1	1,04,117,921	AMY2B	478	rs140978983	1	0	26.1	0	0.021	0.035
1	109,446,750	GPSM2	29501	rs61754640	0.994	0.02	19.3	0.029	0.004	0.022
1	226,125,385	LEFTY2	3122	rs2295418	1	0	16.6	0.024	0.003	0.022
2	69,177,269	GKN2	24588	rs62133344	1	0	18.5	0.035	0.011	0.036
2	70,504,399	PCYOX1	20588	rs34041544	1	0.01	26.4	0.014	0	0.030
2	179,486,345	TTN	12403	rs114331773	1	0	15.7	0	0.024	0.017
2	179,666,982	TTN	12403	rs35683768	0.999	0	15.7	0.024	0.003	0.022
6	76,024,704	FILIP1	21015	rs62415695	1	0.01	15.4	0	0.028	0.009
6	84,904,604	CEP162	21107	rs17790493	1	0	15.9	0	0.025	0.024
7	103,130,222	RELN	9957	rs73714410	0.972	0.02	27.9	0	0.021	0.034
12	124,221,796	ATP6V0A2	18481	rs74922060	1	0.03	23.0	0	0.028	0.010
13	113,750,905	MCF2L	14576	rs140657264	0.999	0	26.6	0	0.024	0.024
16	29,825,022	PRRT2	30500	rs76335820	0.995	0.02	18.4	0.014	0	0.043
17	34,311,387	CCL14	10612	rs16971802	0.974	0.02	16.2	0.011	0.046	0.047
17	37,321,347	ARL5C	31111	rs9912267	1	0	18.6	0.014	0	0.028
18	28,604,374	DSC3	3037	rs35630063	1	0	21.1	0	0.028	0.021
19	56,249,615	NLRP9	22941	rs80009430	1	0	16.0	0.017	0	0.012
20	3,641,868	GFRA4	13821	rs146579049	1	0	18.3	0.017	0	0.017
20	36,954,724	BPI	1095	rs5743523	0.998	0.02	15.5	0.024	0	0.008
22	31,494,813	SMTN	11126	rs80055673	1	0.03	18.7	0.017	0	0.011

**FIGURE 2:**
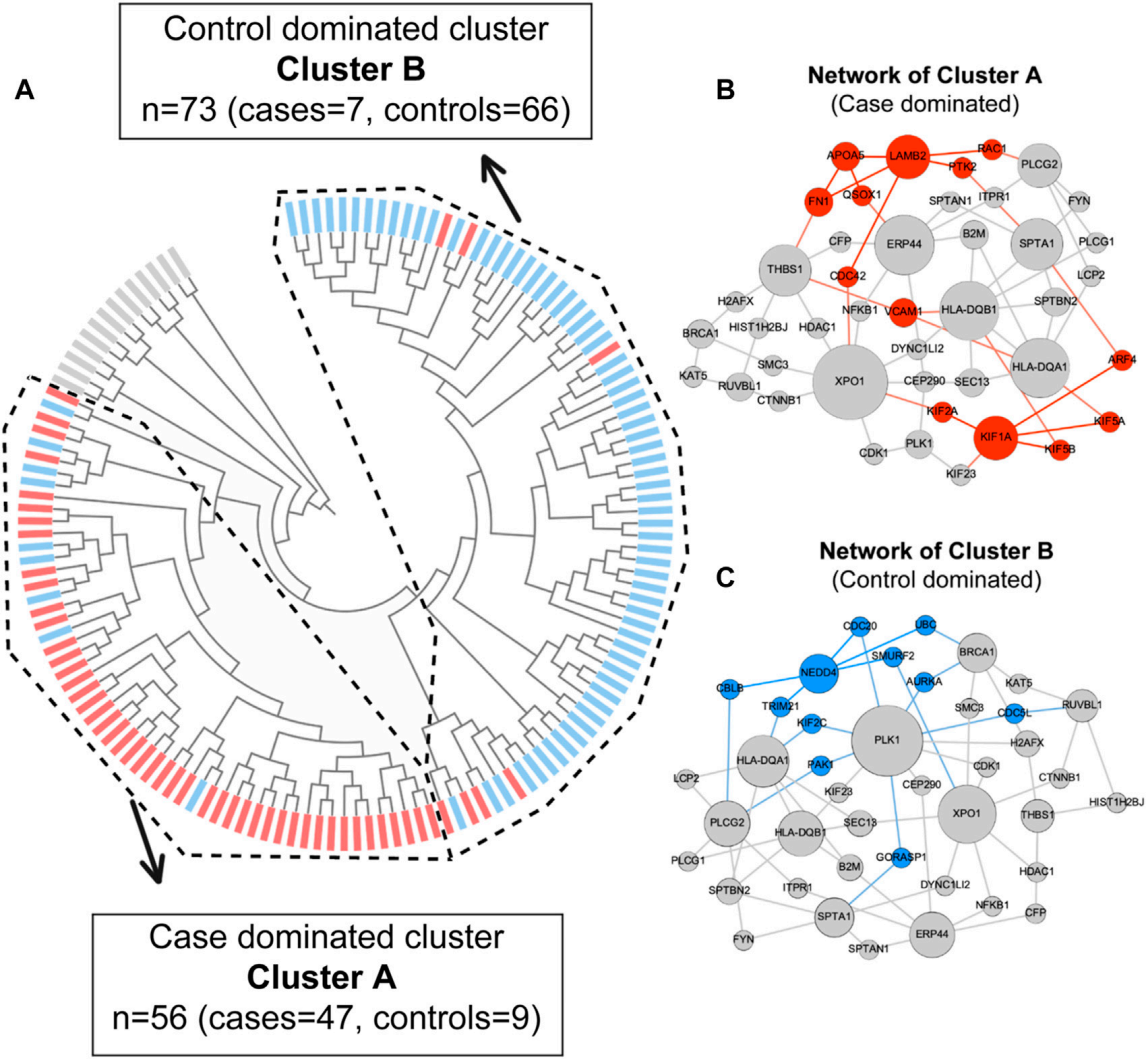
**(A)** Dendrogram shows statistically significant (*p* < 0.05) clusters of patients. Case dominated cluster (Cluster A) and control dominated cluster (Cluster B) are presented by dashed lines. Cases are represented in red and controls are represented in blue color. **(B)** Layered network graphs for the case dominated cluster A are presented. 13 unique genes of cluster A are in red color. **(C)** Layered network graphs for the control dominated cluster B are presented. 11 unique genes of cluster B are in blue color.

*Proteinarium* was used to carry out the multivariate protein protein interaction analysis to identify clusters of patients with shared networks associated with severe preeclampsia. The resulting, circularized dendrogram is shown in Figure 2A (Ciccarelli et al., 2006). Out of the 143 patients sequenced, 129 patients were assigned to two statistically significant clusters. (*p <* 0.0001). The inset in Figure 2A shows the number of cases and controls in each cluster. Cluster A had significantly more cases than controls, containing 47 of the 61 case subjects (Bastian et al., 2009). The layered network for the case-dominated Cluster A is shown in Figure 2B. There are 13 genes which are unique to Cluster A, highlighted in red in the layered network graph. Most have defined functional roles or implications for preeclampsia, Table 3. Cluster B had significantly more controls than cases, including 61 of the 82 subjects. The layered network for Cluster B is shown in Figure 2C. The unique genes from the layered network graph of Cluster B, shown in blue, are listed in Supplementary Table S1.

**TABLE 3:** Unique genes from case dominated cluster (Cluster A). *Genes alphabetically ordered.

Gene name	Gene*	HGNC id	Cluster	Imputed
Apolipoprotein A5	APOA5	17288	A	No
ADP ribosylation factor 4	ARF4	655	A	Yes
Cell division cycle 42	CDC42	1736	A	Yes
Fibronectin 1	FN1	3778	A	Yes
Kinesin family member 1A	KIF1A	888	A	No
Kinesin family member 2A	KIF2A	6318	A	Yes
Kinesin family member 5A	KIF5A	6323	A	Yes
Kinesin family member 5B	KIF5B	6324	A	Yes
Laminin subunit beta 2	LAMB2	6487	A	No
Protein tyrosine kinase 2	PTK2	9611	A	Yes
Quiescin sulfhydryl oxidase 1	QSOX1	9756	A	Yes
Rac family small gtpase 1	RAC1	9801	A	Yes
Vascular cell adhesion molecule 1	VCAM1	12663	A	Yes

We compared the sequence data of the samples not assigned to clusters with those that were assigned and we did not find significant differences in the average depth of coverage. Likewise, there were no significant differences in clinical/phenotypic characteristics when comparing the subjects in the significant clusters with the subjects that were not in these clusters (data not shown).

The comparison of the unique genes from the case and the control dominated clusters revealed a positive separation score, confirming that the layered PPI networks of these two patient subgroups indeed exist in distinct areas of the interactome. We ran GO term analysis using DAVID software on all genes of the network from Cluster A and from Cluster B, Supplementary Table S2 (Huang da et al., 2009b; a). We found significantly enriched biological processes, molecular functions and cellular components based on Bonferroni corrected *p*-value for the case and control dominated networks. Prominent among the biological processes and molecular functions associated with preeclampsia were antigen processing and presentation, cellular movement (axon guidance and microtubules) and T cell receptor signaling.

We previously reported the database for Preeclampsia (dbPEC) which archives a curation-based collection of genes associated with preeclampsia and their association with clinical features and concurrent conditions (Triche et al., 2014). We compared the genes from our univariate analysis and the genes from both case and control dominated layered networks to those in the database. We found two overlapping genes from the univariate gene list (TTN and CCL14) that were included in dbPEC. We also found three overlapping genes from the layered network of Cluster A (FN1, KIF2A, VCAM1). By over representation analysis, Cluster A is significantly enriched for genes previously shown to be associated with preeclampsia in dbPEC (*p* < 0.0033).

## Discussion

Preeclampsia is a life-threatening, multi-system hypertensive disorder of pregnancy, which complicates up to 15% of US deliveries (Chappell and Morgan, 2006; Valenzuela et al., 2012; Bornstein, 2020; Steinthorsdottir et al., 2020). The incidence is increasing (Bornstein, 2020). It is recognized as a leading cause of maternal and fetal morbidity and mortality worldwide (Valenzuela et al., 2012). Preeclampsia is characterized by varying degrees of maternal symptoms including elevated blood pressure, proteinuria and fetal growth restriction (Jebbink et al., 2012). Many clinicians believe that preeclampsia, severe preeclampsia, and early vs late preeclampsia are different disorders (Carreiras et al., 2002; Raymond and Peterson, 2011; American College of et al., 2013). Previously, using bioinformatic methods, we showed that there are discrete gene sets associated with these different phenotypes of preeclampsia (Triche et al., 2014).

We performed whole exome sequencing on women with idiopathic early-onset preeclampsia with severe features and singleton births <34 weeks’ gestation and compared them to term controls with no family history of preeclampsia. We developed *Proteinarium,* a novel multi-sample PPI analysis and visualization tool, to identify clusters of patients with shared protein protein interaction networks (Armanious et al., 2020). Unlike other tools which use the differentially expressed genes or the phenotype associated variants to build a single network for all samples, or clustering models based solely on gene expression, *Proteinarium* builds individual networks for *each* patient based on the STRING database. The similarities between individual PPI networks are then evaluated using a distance metric for clustering the samples. We identified a single, significant, large cluster of patients with a predominance of cases with early-onset, severe features of preeclampsia. We also identified a single control-dominated cluster. The separation test of the unique genes from case and control dominated clusters confirmed that the two subnetworks forming clusters A and B exist in the different regions of the interactome. These results support our hypothesis that the genetic architecture of complex diseases is characterized by clusters of patients that have variants in shared gene networks and provide insights into the genetics of severe preeclampsia.

Several of the unique genes from the case dominated network have very plausible mechanistic connections to preeclampsia. Laminin β2 (LAMB2) is a glomerular basement membrane (GBM) component, required for proper functioning of the glomerular filtration barrier. It has a role in proteinuria (Zhang and Huang, 2012) and serum laminin levels in preeclamptic patients are significantly higher than those in normal pregnancy (Furuhashi et al., 1993). Hypoxia-induced upregulation of Quiescin Sulfhydryl oxidase 1 (QSOX1) and an elevation in intracellular H_2_O_2_ leads to increased apoptosis in the placentae of pregnancies complicated by preeclampsia (Li et al., 2019). QSOX1 protein is found in circulating extracellular vesicles of both preeclampsia and healthy pregnant women (Tan et al., 2014). Fibronectin 1 (FN1) might promote the development of preeclampsia by modulating differentiation of human extravillous trophoblasts, as well as formation of focal adhesions (Brubaker et al., 1992; Auer et al., 2010; Zhao et al., 2017). Vascular Cell Adhesion Molecule 1 (VCAM1) is involved in cellular adhesion and serum concentrations of sVCAM-1 are significantly elevated in both mild and severe preeclampsia (Kim et al., 2004). Invasion of maternal decidua and uterine spiral arteries by extravillous trophoblasts is required for establishment of normal placenta. Human trophoblast migration requires Rac Family Small gtpase 1 (RAC1) and Cell Division Cycle 42 (CDC42) (Grewal et al., 2008). Lower levels were found in preeclampsia samples than in normal term pregnancy samples, and decline significantly in severe preeclampsia (Fan et al., 2016). Protein tyrosine kinase 2 (PTK2) (focal adhesion kinase) is differentially expressed in preeclampsia and reported as among the promising biomarkers for preeclampsia (Sado et al., 2011). In the case-dominated subnetwork we observed Kinesin Family Member 2A (KIF2A) which is upregulated in the preeclamptic placenta (Kobayashi, 2015). Up-regulated genes in the preeclampsia placenta have been shown to be associated with the regulation of diverse cellular processes, including matrix degradation, trophoblast cell invasion, migration and proliferation (Kobayashi, 2015).

There have been several sequencing efforts, including whole genome, whole exome and targeted sequencing, on an array of preeclampsia phenotypes from diverse populations (Johnson et al., 2012; Emmery et al., 2016; Kaartokallio et al., 2016; Thomsen et al., 2017; Gammill et al., 2018; Hansen et al., 2018; Soellner et al., 2018; Glotov et al., 2019; Melton et al., 2019; Steinthorsdottir et al., 2020; Zhang et al., 2020). There is no consensus among the published results in regards to associated genes and variants. Since preeclampsia is a complex, polygenic disease, the lack of a consensus among these univariate comparisons might be expected in these early-stage studies. Among the 20 genes identified in our univariate analysis, only Titin (TTN) was identified in prior studies (Gammill et al., 2018; Zhang et al., 2020). Protein-altering mutations in TTN have been identified in patients with cardiomyopathy and women with preeclampsia are more likely to carry TTN mutations associated with idiopathic cardiomyopathy and peripartum cardiomyopathy (Gammill et al., 2018). Additionally, we found 2 genes, Major Histocompatibility Complex, Class II, DQ Alpha 1 (HLA-DQA1) and Inositol 1,4,5-Trisphosphate Receptor Type 1 (ITPR1) that were reported in previous studies of preeclampsia (Emmery et al., 2016; Hansen et al., 2018). None of these overlapping genes were among the unique genes identified in the shared layered network from the case-dominated cluster. Likewise, no overlapping variants or genes were found in a recent genome-wide association meta-analysis investigating genetic predispositions associated with preeclampsia (Steinthorsdottir et al., 2020).

Our analysis allowed us to identify a cluster of patients with shared PPI networks associated with severe preeclampsia. Within the significant cluster, there were unique imputed genes (RAC1, KIF5B, PTK2, KIF5A, FN1, QSOX1, ARF4, VCAM1, CDC42, KIF2A) that were not among the top 60 seed genes selected by genotype testing. Nonetheless, our approach allowed us to identify these influential genes in the mechanism(s) underlying preeclampsia that would not otherwise have been identified by whole genome univariate variant analysis.

We also examined the unique genes in the network of the control dominated cluster. Proteins in this network are associated with the ubiquitination process. They may serve a role that confers resilience against preeclampsia (Fredrickson and Gardner, 2012; Berryman et al., 2019). Although there are studies showing a relationship with hypertension - ubiquitination process and pregnancy, this still needs further investigation (Fredrickson and Gardner, 2012).

Whole exome sequencing, combined with a novel, multi-sample network analysis, and careful phenotyping contributed to our discovery despite the relatively modest size of our study. Concepts developed from network theory suggest that related diseases involve proteins in similar neighborhoods of the interactome (Menche et al., 2015). Based on these concepts, we hypothesized that the genetic architecture of preeclampsia is described by subgroups of patients with variants in shared genes in specific networks and pathways. We identified a significant subgroup of cases with shared PPI networks associated with severe preeclampsia. We believe that the careful phenotyping resulted in the high percentage of subjects being successfully assigned to significant clusters and the ability to observe distinct separation between the case and control dominated clusters. We acknowledge that genetic variation may not be the sole mechanism for preeclampsia, but rather epigenetic changes or protein conformational disturbances may also play a significant role.

While we were not expecting each patient to appear in a significant cluster and our study included only a modest sample size, we identified a significant subgroup of patients with shared PPI networks associated with severe preeclampsia. In order to leverage the likelihood of genetics discovery, we focused exclusively on women with severe, early-onset preeclampsia. Our analysis was restricted to evaluation of genetic variants in the maternal genome only. Future studies including fetal and/or paternal data will enhance the likelihood of genetic discovery.

Using our unique network analysis, we were able to identify subsets of patients with shared networks, thus confirming our hypothesis about the genetic architecture of preeclampsia. Strict phenotyping of both cases and controls improved the likelihood of identifying these otherwise difficult to find genetic associations. Our network analysis identified genes which were imputed from the interactome and these imputed genes provide insights for severe preeclampsia that may otherwise have not been identified. As such, these are important candidates to include in meta-analyses of genetic associations with preeclampsia. These results provide promise to further our understanding the mechanism underlying complex diseases like preeclampsia.

## Data Availability

The original contributions presented in the study are included in the article/Supplementary Material, further inquiries can be directed to the corresponding author.
